# Paving the Way for Integrated Advanced Heart Failure and Adult Congenital Heart Disease Training

**DOI:** 10.1016/j.jacadv.2025.101586

**Published:** 2025-02-06

**Authors:** William H. Marshall V., Ryan D. Byrne, Andrew Civitello, William J. Dreyer, Peter R. Ermis, Brent C. Lampert, Lauren T. Lastinger, Deipanjan Nandi, Joseph A. Spinner, Curt J. Daniels

**Affiliations:** a Division of Cardiology, Department of Internal Medicine, The Ohio State University Wexner Medical Center, Columbus, Ohio, USA; b The Heart Center, Nationwide Children's Hospital, Columbus, Ohio, USA; c Adult Congenital Heart Program, Department of Pediatrics, Section of Cardiology, Texas Children's Hospital, Baylor College of Medicine, Houston, Texas, USA; d Division of Cardiothoracic Transplantation and Circulatory Support, Michael E. DeBakey Department of Surgery, Baylor College of Medicine, Houston, Texas, USA; e Department of Cardiopulmonary Transplantation and the Center for Cardiac Support, Texas Heart Institute, Houston, Texas, USA; f Department of Pediatrics, Section of Cardiology, Baylor College of Medicine/Texas Children's Hospital, Houston, Texas, USA

**Keywords:** adult congenital heart disease, curricular development, fellow-in-training, heart failure

Heart failure (HF) is the leading cause of morbidity and mortality in adults with congenital heart disease (CHD).^1^ However, compared to patients with acquired HF, the adult CHD (ACHD) population of patients with HF is unique and includes those with complex forms of CHD, systemic right ventricles, and single ventricle anatomy with Fontan palliation. Additionally, patients with ACHD and HF are less likely to be treated with advanced HF therapies, including durable mechanical circulatory support or heart transplant.^2^ This may be in part due to the under-recognition of the unique indications for transplant in the ACHD population, and lack of dedicated training in ACHD HF during adult advanced HF transplant cardiology (AHFTC) fellowship.^3^ While general Accreditation Council for Graduate Medical Education (ACGME) milestones in HF training for all ACHD fellows-in-training (FITs) have been outlined, given the growing number of ACHD HF patients with medical, anatomic, and surgical complexity, there is a need to train an ACHD HF physician workforce to provide tailored care for this emerging population. Though there have been prior calls for cross disciplinary training^3^ and “sub-subspecialization” in an area of ACHD during training,^4^ to date no clear roadmap exists for training in ACHD HF. It is possible to pursue a third fellowship in HF (Figure 1A, top), and a specific ACHD “circulatory failure” fellowship has been proposed;^5^ however, these all require an additional year of training in an already lengthy pathway. Other cardiology subspecialties have formalized competency-based training pathways which can reduce training time,^6^ though a similar pathway for HF and ACHD does not exist. Authors W.H.M.V. and R.D.B. have had the opportunity to integrate HF training within their ACHD training time (Figure 1A, bottom) and would like to share those pathways and disseminate developed curricula to help others aspiring to train in this important and developing field.

**Figure 1:**
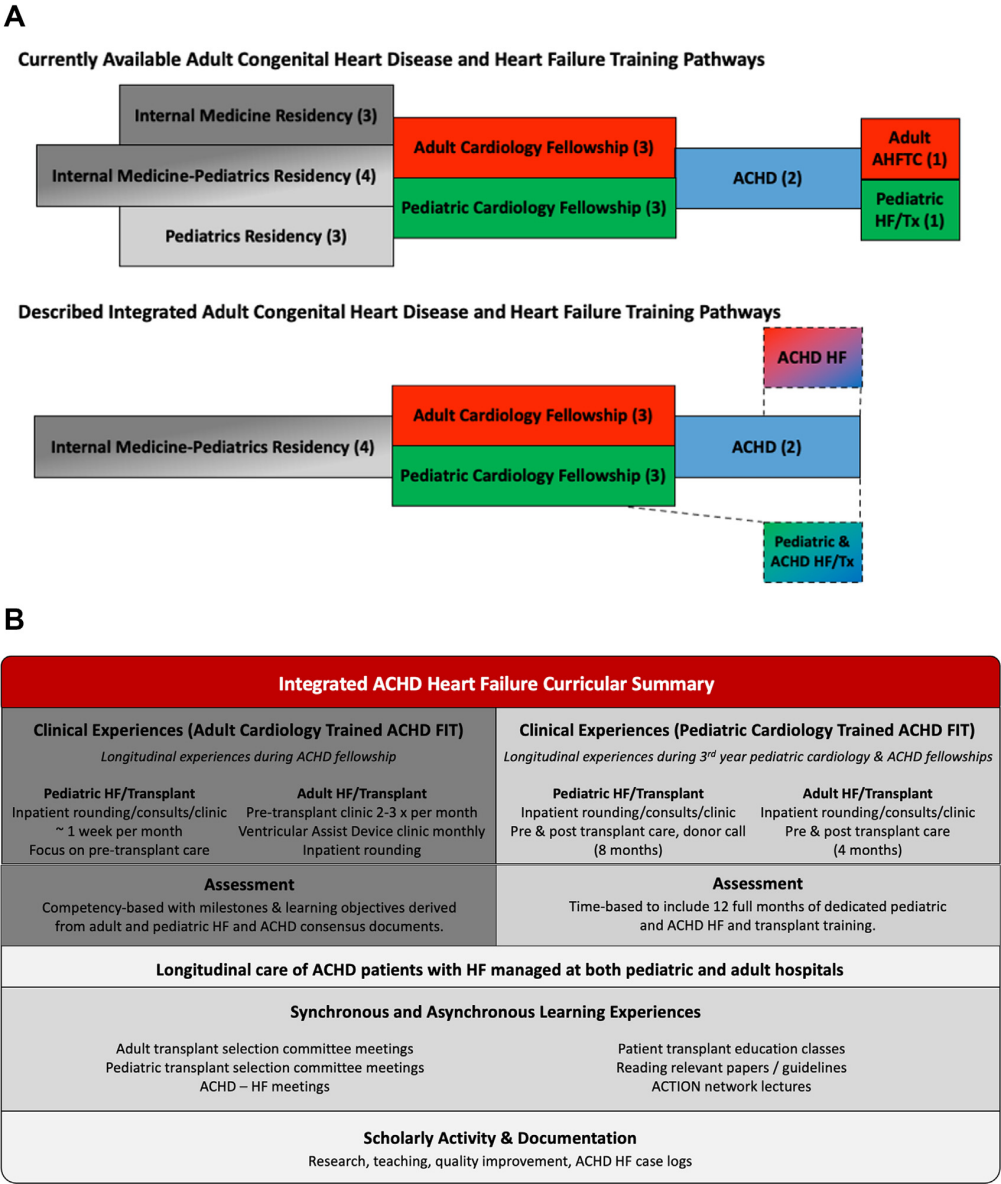
**Integrated Adult Congenital Heart Disease and Advanced Heart Failure Training** (A) Current and described pathways in ACHD and HF training and (B) integrated ACHD HF curricular summary. ACHD = adult congenital heart disease; ACTION = advanced cardiac therapies improving outcomes network; AHFTC = advanced heart failure and transplant cardiology; FIT = fellows-in-training; HF = heart failure; Tx = transplant. Panel A, years of training indicated in parentheses.

Although competency-based milestones exist for ACHD and AHFTC training, due to time-based requirements, it is not possible to become certificated in ACHD and AHFTC without completing both fellowships (Figure 1A, top). While some may chose this pathway, another solution is to train a physician workforce with complementary skillsets, where an ACHD physician can serve as a “bridge” between the ACHD and HF/transplant teams to optimize medical therapy and streamline the assessment of ACHD patients for advanced HF therapies. To accomplish this, author W.H.M.V. and colleagues developed and implemented a competency-based curriculum for the adult cardiology-trained ACHD FIT, which is integrated into ACHD fellowship and focuses on the evaluation and treatment of ACHD patients with advanced HF (Figure 1A, bottom). Described in detail previously,^7^ this curriculum was designed utilizing the ACGME milestones for both the AHFTC and ACHD training as a framework to design learning objectives, which were adapted for the ACHD FIT by removing post-transplant care and integrating learning objectives from the AHFTC Clinical Competency Statement and Pediatric Heart Failure Core Competency Curriculum.^8^^,^^9^ Clinical rotations in pediatric and adult HF and other curricular activities were designed with experts in pediatric and adult HF and ACHD, such that the ACHD FIT has exposure to advanced HF therapies for both acquired and ACHD HF and can follow patients longitudinally at both adult and pediatric institutions. Case logs, conferences, asynchronous learning, and catheterization lab time all assured adequate exposure to ACHD HF to achieve the learning objectives (Figure 1B). The focus of this pathway was not on post-transplant care, acknowledging that all the requirements for AHFTC fellowship do not have to be pursued during ACHD HF training. As an early career physician, through clinical care and participation in both the pediatric and adult HF transplant selection committees, this approach has allowed the author (W.H.M.V.) to advance ACHD patients through the transplant evaluation in a system where patients are evaluated and listed at either the pediatric or adult HF/transplant program depending on anatomy, physiology, and comorbidities. For patients with ACHD and HF assessed by the pediatric HF team, this position allows the author to fill in the gaps in adult care for a comanagement paradigm during the transplant education, evaluation, listing, and (often prolonged) wait period for these patients. When patients are assessed for advanced HF therapies by the adult HF team, having someone in this position has helped to identify and educate others on specific ACHD transplant indications, discuss issues related to congenital anatomy and physiology, and perform cardiac catheterizations for hemodynamic assessment of patients with congenital anatomy (residual shunts, d-transposition of the great arteries after atrial switch, congenitally corrected transposition of the great arteries, etc.). For patients with ACHD who have undergone placement of a durable ventricular assist device, we have adopted a comanagement strategy with the HF teams such that their residual congenital anatomic issues are assessed as needed. Though it is early in the process, through clinical evaluation, cross institutional communication, and facilitation of regular multidisciplinary meetings, thus far the assessment of patients with ACHD for advanced HF therapies has become more streamlined at our institutions.

There are both benefits and limitations to this pathway. One benefit of a competency-based curriculum is that prior experience in HF training can be accounted for, such that some FITs may require less dedicated time to achieve the same competencies. However, this may not be generalizable to other ACHD programs given the need for an established ACHD program with both adult and pediatric HF/transplant programs. Additionally, though the curriculum was designed for the adult cardiology trained ACHD FIT, many who pursue training in ACHD first complete a fellowship in pediatric cardiology. While the curriculum^7^ could be altered for the pediatric cardiology trained ACHD FIT, an alternative pathway could be pursued, as described below.

Author R.D.B. completed pediatric cardiology fellowship followed by ACHD fellowship at a high-volume ACHD and pediatric HF/transplant center and had the opportunity to participate in a novel curriculum, encompassing the final year of categorical pediatric cardiology fellowship and ACHD fellowship (Figure 1A, bottom). This included 12 total months of dedicated adult and pediatric HF/transplant clinical time, structured to provide an experience similar to that of a dedicated advanced pediatric HF/transplant FIT (Figure 1B). Clinical experiences included the management of end-stage HF and CHD; mechanical circulatory support; pretransplant care including heart transplant recipient education and evaluation; peri-transplant care, including waitlist management, donor offer evaluations, organ procurement and preservation, and peri-operative care; and post-transplant care, including surveillance and management of complications such as acute rejection, coronary allograft vasculopathy and graft dysfunction, and management of infections related to immunosuppression. Altogether, these experiences created a foundation of knowledge to ensure aptitude in managing HF/transplant in both the pediatric and ACHD populations. ACHD HF training via a pediatric cardiology pathway allows for the trainee to be well equipped to care for this complex cohort of patients, no matter how an institution is structured. At an adult hospital, the CHD-specific HF experiences and perspective that this training pathway provides can help that institution better care for ACHD HF patients with end-stage physiology during the transplant evaluation, waitlist, and peri-operative time periods. At a children's hospital performing ACHD heart transplants, this training pathway could provide age-appropriate support to help guide post-ACHD transplant management. In either scenario, dedicated pediatric HF/transplant time may also be possible if the institution is able to accommodate such an interest.

There are both benefits and limitations to this pathway. As advanced fellowship in pediatric HF/transplant is not an ACGME accredited fellowship, the equivalency in training time is able to be accomplished (most likely in a noncontiguous fashion) over the course of pediatric categorical and ACHD fellowships, thereby avoiding an additional year of fellowship in an already lengthy ACHD training pathway. Though there is no board certification required for pediatric HF/transplant, a high level of expertise in CHD HF and transplant medicine must still be accomplished, and credibility still demonstrated through documentation of patient experiences with a training log and, if needed, letters of recommendation. Ultimately, as this training pathway departs from the standard advanced training curriculum that many hiring institutions rely on for educational integrity, early and transparent conversations with institutions while seeking a faculty position are paramount. More practically, this pathway may not be generalizable to those without the background training of internal medicine-pediatrics to allow ample ACHD elective time, or for pediatric cardiology FITs who do not identify an interest in ACHD HF early enough to complete some pediatric HF/transplant training time during categorical fellowship. Similar to the adult ACHD HF training pathway, this training is not possible without a robust ACHD and pediatric HF/transplant program in place. Finally, neither of the above training pathways are possible without the support of mentors and educators within ACHD and both pediatric and adult HF/transplant, allowing for the flexibility and advocacy during ACHD fellowship to pursue this training. Both authors experienced unequivocal commitment and open-mindedness from the ACHD, adult and pediatric HF/transplant programs to ensure the rotations and experiences were not just fulfilled, but also deeply educational.

The prevalence of adults with CHD will continue to rise over the next several decades,^10^ leading to greater ACHD HF needs in a system where the number of patients with ACHD and HF exceeds the current care models and patient care is already affected. To care for and improve outcomes for this anatomically and physiologically complex population of adults with CHD and HF, we are compelled and obligated to develop a new subspecialty field—ACHD HF—with subspecialists trained in ACHD, and pediatric and adult HF. To meet this demand, unique training pathways are proposed with focus on achieving specific ACHD HF competencies and milestones. In this way, ACHD HF cardiologists and this sub-subspecialty field can develop further educational goals and training pathways, stimulate and advance clinical excellence, outline quality metrics and initiatives, and develop a research agenda. The training pathways described above are a step toward an ACHD HF agenda to improve patient outcomes.

## Funding Support and Author Disclosures

The authors have reported that they have no relationships relevant to the contents of this paper to disclose.
